# Twisted bilayer systems: a versatile platform for exploring emergent quantum phenomena

**DOI:** 10.1093/nsr/nwag057

**Published:** 2026-01-28

**Authors:** Tingxin Li, Ding Zhang, Qikun Xue

**Affiliations:** State Key Laboratory of Micro-nano Engineering Science, School of Physics and Astronomy, Shanghai Jiao Tong University, China; State Key Laboratory of Low Dimensional Quantum Physics and Department of Physics, Tsinghua University, China; Beijing Academy of Quantum Information Sciences, China; State Key Laboratory of Low Dimensional Quantum Physics and Department of Physics, Tsinghua University, China; Beijing Academy of Quantum Information Sciences, China; State Key Laboratory of Quantum Functional Materials, Department of Physics, and Guangdong Basic Research Center of Excellence for Quantum Science, Southern University of Science and Technology, China

The assembly of two atomically thin van der Waals materials with a relative twist angle or lattice mismatch naturally creates a moiré superlattice. When the twist angle or lattice mismatch is small, the resulting moiré periodicity can far exceed the underlying atomic lattice constants. This leads to the formation of significantly narrowed electronic minibands compared with those of the parent materials [1]. Such ‘moiré engineering’ not only enhances the correlation effects but also facilitates non-trivial band topology and offers unprecedented tunability via electric fields, carrier densities and pressure, among others. Since the discovery of correlated electronic states and unconventional superconductivity in magic-angle twisted bilayer graphene (MATBG) [2,3], various moiré systems have established themselves as a powerful and versatile platform for investigating emergent quantum phenomena driven by strong correlations and non-trivial topology. A notable experimental breakthrough recently in this context is the observation of fractional quantum anomalous Hall effects in twisted moiré systems [4–8]. This *NSR* Special Topic brings together nine articles that collectively illustrate how twisted bilayer systems have developed into a rich and highly tunable material platform for exploring correlated and topological quantum matter.

The review paper by Li *et al.* [9] summarizes the rapidly expanding landscape of quantum phases in twisted transition metal dichalcogenide (TMDc) homobilayers. The authors elucidate how the skyrmionic texture of layer pseudospins generates a giant emergent magnetic field, leading to the formation of topological moiré flat bands with ideal quantum geometry in twisted homobilayer TMDc. They highlight the consequent realization of a cascade of interaction-driven phases—ranging from integer and fractional quantum anomalous Hall insulators to zero-field composite Fermi liquids and unconventional superconductivity—all tunable by twist angle, displacement field and carrier density. They also discuss open questions and outline promising directions for future research. In a perspective article, Mak and Shan [10] discuss how TMDc moiré semiconductors can serve as highly tunable solid-state simulators of the Hubbard model. They outline the realization of both triangular and honeycomb lattice Hubbard physics in TMDc hetero- and homobilayers, highlighting the continuous control over the ratio of onsite Coulomb repulsion *U* to bandwidth *W*. They give an overview of the experimental observations of a rich phase diagram, encompassing Mott insulators, generalized Wigner crystals, kinetic magnetism and, most recently, superconductivity emerging adjacent to a bandwidth-tuned Mott transition.

The exploration of novel topological quantum phases has become one of the central focuses in current moiré physics research. In MATBG aligned with hexagonal boron nitride, Zhang *et al.* [11] report a complete cascade of zero-field Chern insulators at all odd-integer moiré fillings (*ν* = ±1, ±3), establishing the full topological sequence in MATBG. Moreover, they reveal symmetry-broken Chern insulators at a fractional filling *ν* = −7/2 and observe magnetic-field-stabilized incommensurate Chern insulator states extending from *ν* = −3. Liu *et al.* [12] investigate the microscopic electronic structure of twisted bilayer MoTe_2_ using scanning tunneling microscopy and spectroscopy. Their experiments reveal real-space localization of flat-band wavefunctions, electric-field-driven topological transitions, and the formation of Wigner molecular crystals with emergent Kagome lattice geometry at commensurate moiré fillings, providing microscopic insight into the topological flat bands formed in twisted bilayer MoTe₂. Going beyond electronic ground states, Zhou *et al.* [13] theoretically demonstrate that topological properties can be inherited by collective spin excitations in twisted TMDc, predicting itinerant magnons and spin excitons with non-trivial topology and electrically tunable thermal Hall responses. Extending the notion of emergent topology to composite quasiparticles, Wang and Yao [14] uncover a genuine non-Abelian lattice gauge field governing biexcitons in twisted bilayer MoTe₂, leading to an effective Kagome lattice and enabling non-Abelian Aharonov–Bohm interference and controlled generation of entangled states.

Two research articles emphasize the crucial role of lattice reconstruction, strain and structural inhomogeneity in twisted bilayer systems. Using scanning tunneling microscopy, Ouyang *et al.* [15] investigate marginally twisted bilayer graphene at extremely small twist angles and reveal strain-induced transitions between distinct types of domain walls with characteristic electronic signatures. From a theoretical perspective, Li *et al.* [16] develop an analytical framework that quantitatively relates twist angle to strain, stress, rotation fields and energetics in twisted 2D materials, providing a mechanical foundation for understanding how atomic-scale reconstruction influences electronic and topological properties.

Twisted bilayer systems also open new avenues for investigating high-temperature superconductivity. In twisted cuprate Josephson junctions, Zhu *et al.* [17] systematically study the AC Josephson effect and demonstrate that fractional Shapiro steps can be manipulated through magnetic-field training and current annealing. Their results show that such fractional steps are not uniquely tied to topological superconductivity, highlighting both the opportunities and the challenges in interpreting experimental signatures in twisted high-temperature superconductors.

Collectively, these articles demonstrate that twisted bilayer systems provide an ideal material platform where geometry, topology and strong correlations intertwine. By combining powerful theoretical frameworks with advanced experimental probes, these works deepen our understanding of quantum matter in moiré superlattices and point toward future directions, including the controlled realization of exotic superconducting states, non-Abelian excitations and scalable quantum simulators. We hope this Special Topic will inspire further interdisciplinary efforts and stimulate continued exploration of twisted bilayer quantum materials.
